# Synergistic and Antagonistic Effects of Chemical Pollutants and Parasitic Fungi on Cyanobacterial Metabolism

**DOI:** 10.1002/tox.70073

**Published:** 2026-03-08

**Authors:** Erika Berenice Martínez‐Ruiz, Jutta Fastner, Stephanie Spahr, Justyna Wolinska

**Affiliations:** ^1^ Department of Evolutionary and Integrative Ecology Leibniz Institute of Freshwater Ecology and Inland Fisheries (IGB) Berlin Germany; ^2^ German Environment Agency Section Protection of Drinking Water Resources Berlin Germany; ^3^ Department of Ecohydrology and Biogeochemistry Leibniz Institute of Freshwater Ecology and Inland Fisheries (IGB) Berlin Germany; ^4^ Institute of Biology Freie Universität Berlin Berlin Germany

**Keywords:** biomarkers, cigarette butts, environmental parasitology, integrated biomarker response, metolachlor

## Abstract

Freshwater ecosystems are increasingly impacted by anthropogenic pollutants, including the widely used herbicide metolachlor (MET) and cigarette butt (CB) litter. Parasites represent an additional biotic stressor that can modulate pollutant effects on their hosts. Cyanobacteria are key primary producers in aquatic ecosystems, and alterations in their metabolism can cascade through trophic webs, affecting ecosystem functioning. However, the metabolic responses of cyanobacteria to simultaneous exposure to pollutants and parasites remain poorly understood. In this study, we investigated the metabolic response of the toxigenic cyanobacterium *Planktothrix agardhii* using a multi‐biomarker approach under simultaneous exposure to abiotic (MET and CB leachate) and biotic (the chytrid parasite *Rhizophydium megarrhizum*) stressors. Co‐exposure to MET and chytrids led to a synergistic effect, inducing oxidative stress, primarily countered by non‐enzymatic mechanisms, whereas MET alone had no measurable effect. In contrast, CB leachate alone induced oxidative stress, but this effect was mitigated when cyanobacteria were also infected by chytrids, indicating an antagonistic interaction. This study demonstrates the complexity of cyanobacterial metabolic responses to interacting biotic and abiotic stressors and highlights the importance of incorporating host–parasite interactions in ecotoxicological assessments. Metabolic alterations induced in toxigenic cyanobacteria by multiple stressors may influence bloom formation and trophic energy transfer, with broader ecological implications in freshwater ecosystems.

## 1 Introduction

Cyanobacteria are essential primary producers in aquatic systems, forming the base of most aquatic trophic webs alongside other phytoplankton. Eutrophication is a major driver of cyanobacterial proliferation in freshwater systems [1], and climate change can further exacerbate this process [2]. Together, these conditions often lead to harmful cyanobacteria blooms, which can deplete oxygen [3] and produce metabolites that pose significant risks to humans and other organisms [4, 5].

Cyanobacterial growth is regulated by both bottom‐up factors, such as nutrient availability [1], and top‐down interactions, including parasitism by chytrids [6]. Chytrids, zoosporic fungi from the phylum Chytridiomycota, are common parasites of phytoplankton, including cyanobacteria [7]. These infections are lethal, delaying or suppressing bloom formation [6, 8, 9], while also creating alternative trophic links between primary producers and grazers [10, 11, 12]. Thus, both chytrids and cyanobacteria play crucial roles in aquatic ecological and evolutionary processes.

Cyanobacteria, chytrids, and other aquatic organisms are increasingly affected by water pollution from agricultural runoff and insufficiently treated domestic and industrial wastewater [13, 14]. Common pollutants include chemicals such as metals, pharmaceuticals, and herbicides like metolachlor (MET), as well as solid wastes like cigarette butts (CBs), which release various chemicals into the water. MET, a widely used chloroacetanilide herbicide, is applied at an estimated rate of 100 000 tons per year [15]. In surface waters, concentrations typically range from ng L^−1^ to low μg L^−1^ but can reach up to 400 μg L^−1^ [16, 17, 18, 19, 20, 21]. CBs are among the most littered items worldwide, with around 4.5 trillion improperly discarded annually [22, 23, 24, 25]. Their density in urban areas ranges from 1 to 38 CB per m^2^ [26, 27, 28], posing environmental concerns due to the release of microplastics [29] and chemicals such as nicotine [26, 30].

The combined effects of biotic and abiotic stressors, such as parasites and pollutants, on aquatic organisms have gained attention in recent years [31, 32]. Parasites may exacerbate or mitigate pollutant effects through synergistic and antagonistic interactions, depending on the host–parasite relationship and pollutant type [31]. In previous studies, we found that MET promoted cyanobacterial growth in the absence of chytrids, but this effect disappeared under co‐exposure [33], suggesting greater stress on cyanobacterial cells under combined exposure. In contrast, CB leachate inhibited cyanobacterial growth in uninfected cultures but promoted it in the presence of chytrids to levels comparable to uninfected control cultures [34]. This finding indicated that chytrid infection can mitigate the negative effects of CB leachate. However, the underlying metabolic responses remain unknown.

Biomarkers are biochemical indicators that reflect metabolic changes in organisms in response to stimuli, such as pollutants. These markers provide valuable insights into the metabolic effects, complementing growth‐based assessments and helping elucidate toxicity mechanisms. Both MET and CB leachate can disrupt cellular metabolism: MET exposure has been linked to increased lipid peroxidation [35, 36], elevated cyanotoxin levels, and overexpressed toxin‐synthesis genes [37, 38], whereas CB leachate has been shown to decrease protein levels and increase lipid content and peroxidation [39]. However, information on the effects of pollutants on bloom‐forming, toxigenic cyanobacteria, particularly under simultaneous biotic and abiotic stress, is still limited.

As growth measurement alone is not enough to understand the full impact of pollutants on phytoplankton such as cyanobacteria, complementary biomarker analyses are needed to reveal metabolic alterations and toxicity mechanisms. Multi‐biomarker approaches have been used to assess the effect of stressors. However, integrating multiple biomarker responses into a meaningful interpretation remains challenging. Different tools have been developed to summarize changes across biomarkers into a single value reflecting an organism's overall physiological stress, regardless of individual biomarker direction. One such tool is the Integrated Biomarker Response version 2 (IBRv2) [40].

Here, we investigated how chytrid parasites and anthropogenic pollutants (MET and CB leachate) affect the metabolic responses of cyanobacteria using a multi‐biomarker approach and the IBRv2. Specifically, we assessed macromolecule content, oxidative stress markers, and microcystin production in both uninfected and infected cyanobacteria. These biomarkers were selected based on previously reported effects of MET and CB leachate in aquatic organisms and their relevance for assessing metabolic stress.

Based on our earlier findings, we hypothesized that: (1) MET does not alter the biomarker responses of uninfected cyanobacteria; (2) co‐exposure to MET and chytrids enhances the integrated biomarker response compared to MET alone due to synergistic stress; (3) CB leachate induces oxidative stress and alters macromolecular content and cyanotoxin production in uninfected cells; and (4) co‐exposure to CB leachate and chytrids reduces the integrated biomarker response relative to CB leachate alone, indicating antagonistic interactions.

## 2 Materials and Methods

### 2.1 Cyanobacteria and Chytrids Culturing Conditions

The host–parasite system used in this study consisted of the toxigenic filamentous cyanobacterium *Planktothrix agardhii* strain NIVA‐CYA630 (monoclonal, non‐axenic) and its obligate chytrid parasite *Rhizophydium megarrhizum* strain Chy‐Kol2008 [41]. *R. megarrhizum* infections are lethal to *P. agardhii* [41].

Cyanobacterial cultures were routinely maintained in Z8 medium [42] at 16°C under continuous light (20 μmol photons m^−2^ s^−1^). Chytrids were cultivated by transferring zoospores to uninfected cyanobacterial cultures every 3 weeks under the same conditions. Both organisms were maintained under identical temperature and light regimes throughout the experiment.

### 2.2 Exposure Experiment

We assessed the effects of biotic (chytrid parasites) and abiotic (chemicals) stressors on *P. agardhii* . The concentrations of MET and CB leachate, as well as the sampling time point, were chosen based on previous studies to ensure high cyanobacterial biovolume, active chytrid infection, minimal filament mortality, and a high likelihood of detecting biomarker responses [33, 34].

The experiment consisted of 30 experimental units in a 2 × 3 factorial design, with two infection conditions (infected and uninfected cyanobacteria) and three pollutant treatments (negative control, 100 μg L^−1^ MET and CB leachate corresponding to 1 CB L^−1^), with five replicates per treatment. The experiment was conducted in Falcon 50 mL cell culture bottles with a final volume of 30 mL.

S‐metolachlor (MET, CAS 87392‐12‐9, purity 99.1%) was purchased from Santa Cruz Biotechnology Inc. A 3 mg L^−1^ stock solution was prepared in sterile Milli‐Q water and stored at 4°C. The CB leachate stock solution was prepared as described in Guttman et al. [34] with a concentration equivalent to 100 CB L^−1^. Briefly, self‐rolled CBs were soaked in distilled sterile water and kept in the dark with gentle agitation for 24 h. The resulting solution was sequentially filtered with 30 μm and 5 μm nylon mesh filters, and finally through 1.2 μm glass fiber filters to avoid rapid clogging.

To produce chytrid zoospores, an exponentially growing cyanobacterial culture was infected 10 days prior to the experiment. Afterwards, the infected culture was filtered through a sterile 5 μm nylon mesh and 3 μm polycarbonate membrane [43]. The resulting zoospore suspension was microscopically examined to confirm the absence of cyanobacterial filaments. Zoospore density was quantified using a Sedgewick Rafter chamber under an inverted microscope (Nikon Ti Eclypse).

Cyanobacterial cultures were maintained as exponentially growing semi‐continuous cultures for 2 weeks before the experiment. The optical density (OD_750nm_) was adjusted to 0.05 three times per week and again on the day of exposure before adding MET or CB leachate. This OD corresponds to approximately 10^4^ filaments mL^−1^. Eight 130‐mL cultures were incubated; four were infected with 750 zoospores mL^−1^. Infected cultures were incubated for 6 days to allow infection to be established. After 6 days, infected and uninfected cultures were pooled separately to obtain comparable biomass and homogeneous infection levels within each group, while ensuring a heterogeneous population of cyanobacterial filaments and chytrids. The pooled cultures were then distributed into independent experimental units. All units were incubated for 5 days under the conditions described in Section 2.1.

### 2.3 Sample Collection and Processing

To determine total and intracellular microcystins (MCs), 1 mL of culture was collected from each experimental unit on day 5. The samples were frozen–thawed twice and sonicated for 10 min. To measure extracellular MCs, 1 mL of culture was filtered through a 0.7 μm glass fiber filter (GF/F Grade, Whatmann). Both sonicated extracts and filtrates were further filtered (PTFE, 0.45 μm, Waters) before analysis. All samples were blinded and randomized.

To quantify additional biomarkers, the remaining culture volume (23 mL) was filtered through 0.7 μm glass fiber filters (GF/F Grade, Whatmann) on day 5. Biomass was recovered under sterile conditions, resuspended in 1 mL of phosphate buffer (PBS, pH 7.4), and transferred to a 1.5 mL centrifuge tube. The biomass was washed three times with PBS by centrifugation (15 000*g*, 5 min, room temperature). After the final wash, cells were resuspended in 1 mL PBS and disrupted by ultrasonication (three 30‐s cycles, 35% amplitude, 0.5 cycles; Hielscher Ultrasonics). The samples remained on ice during ultrasonication and between cycles to prevent overheating. This protocol ensures the disruption of over 90% of cyanobacterial and chytrid cells (data not shown).

One tablet of cOmplete Mini EDTA‐free protease inhibitor cocktail (Roche) was added per 10 mL of PBS used for ultrasonicated samples. After disruption, samples were separated into two fractions. One fraction was centrifuged at 9000*g* for 20 min at 4°C, and the supernatant (cell‐free extract) was collected and used for protein content and enzymatic determinations. The second fraction (crude extract) was not centrifuged and was used to determine thiobarbituric acid reactive substances (TBARS) and lipid content. Sample identities were randomized before analysis.

To measure biovolume and parasite fitness proxies, 1 mL of culture was collected, fixed with acid Lugol and stored at 4°C. All sample identities were blinded and randomized. For chemical analyses, 1 mL of liquid culture was filtered through 0.22 μm regenerated cellulose syringe filters (Chromafil Xtra RC‐20/13, Macherey‐Nagel) to remove bacteria and fungi. Samples were collected on days 0 and 5 and stored at 4°C until analysis.

### 2.4 Recorded Parameters

#### 2.4.1 Biomarkers

##### 2.4.1.1 Proteins

Protein content was quantified using the Bradford method [44]. Measurements were performed in 96‐well transparent flat‐bottom plates adding 95 μL of Bradford reagent and 5 μL of cell‐free extract per well. Absorbance was measured at 595 nm using bovine serum albumin as standard. Each sample was quantified six times as technical replicates. Protein content was adjusted to the total biovolume and expressed as mg mm^−3^.

##### 2.4.1.2 Lipids

Lipid content was determined using the sulfo‐phospho‐vanillin method [45]. Lipids were first extracted by adding 300 μL of a chloroform: methanol solution (2:1, v/v) to 100 μL of crude extract [46]. After mixing by inversion, 250 μL of water was added to induce phase separation. The lower phase was transferred to a 1.5 mL centrifuge tube and heated at 80°C to evaporate the solvent. Following evaporation, 25 μL of concentrated sulfuric acid was added, and samples were heated for 10 min at 80°C. Samples were then cooled to room temperature, and 250 μL of phosphor vanillin reagent (1.2 g L^−1^ vanillin in 68% phosphoric acid) were added. After gentle mixing and incubation on ice for 5 min, 100 μL of each sample were transferred to a 96‐well flat‐bottom transparent microplate, and absorbance was measured at 525 nm. Canola oil was used as standard [47]. Each sample was quantified three times as technical replicates. Lipid content was adjusted to the total biovolume and expressed as mg mm^−3^.

##### 2.4.1.3 Microcystins (MCs)

MCs were quantified by liquid chromatography–tandem mass spectrometry (LC–MS/MS) using an Agilent 1290 series HPLC system (Agilent Technologies, Waldbronn, Germany) coupled to an API 5500 QTrap mass spectrometer (AB Sciex, Framingham, MA, USA) equipped with a turbo‐ion spray interface. Samples were separated at 30°C using a Purospher STAR RP‐18 end‐capped column (30 mm × 4 mm, 3 mm particle size, Merck, Germany) with a mobile phase consisting of 0.5% formic acid (A) and acetonitrile with 0.5% formic acid (B) at a flow rate of 0.5 mL min^−1^ with a gradient program (0 min 25% B, 10 min 70% B, 11 min 70% B). The injection volume was 10 μL. Identification and quantification of MCs ([Asp3]‐MC‐RR, MC‐RR, MC‐YR, [Asp3]‐MC‐LR, MC‐LR, MC‐LW, MC‐LF, and MC‐LA; standards purchased from Novakits, France) were performed in the Multiple Reaction Monitoring (MRM) mode. The detection limits of the different congeners ranged from 0.05 to 0.3 μg L^−1^. Intracellular MC (MCi) concentration was estimated as the difference between total MC (MCt) and extracellular MC (MCe). Among the MCs analyzed, we exclusively detected [Asp3]‐MC‐RR, [Asp3]‐MC‐LR, and an additional MC variant present at a concentration too low for precise identification. To maintain clarity throughout the text, these will be referred to as DMC‐RR, DMC‐LR, and MC‐X, respectively.

##### 2.4.1.4 Thiobarbituric Acid Reactive Substances

TBARS, an indicator of lipid peroxidation levels, were quantified according to Ohkawa et al. [48]. First, 400 μL of the reaction solution (0.375% 2‐thiobarbituric acid, 0.5% sodium dodecyl sulfate, and 9.375% acetic acid pH 4) was added to 100 μL of crude extract. The mixture was incubated at 95°C for 1 h. After cooling on ice, samples were centrifuged at 1200 *g* for 5 min. Finally, 150 μL of each sample was transferred to a 96‐well microplate, and absorbance was measured at 532 nm. 1,1,3,3,‐tetramethoxypropane (TMOP) was used as standard. Each sample was quantified four times as technical replicates. Since other molecules besides malondialdehyde and 4‐hydroxyhexenal (the primary products of lipid peroxidation) can also react with thiobarbituric acid, the measurements are referred to as TBARS levels. The TBARS content was adjusted to the total biovolume and expressed as μmol mm^−3^.

##### 2.4.1.5 Superoxide Dismutase (SOD) Activity

SOD activity was quantified by a colorimetric technique using the SOD activity assay kit (Sigma‐Aldrich). Briefly, 20 μL of cell‐free lysate and 160 μL of working solution were added to a 96‐well plate. The reaction was initiated by adding 20 μL of xanthine oxidase working solution to each well, and the plate was incubated at 20°C–25°C for 30 min. Absorbance was measured at 450 nm. SOD supplied by the manufacturer was used as standard. The SOD activity was adjusted to the total biovolume and expressed as units mg^−1^ of protein.

##### 2.4.1.6 Glutathione ***S***‐Transferase (GST) Activity

GST activity was quantified using a modified version of the method described by Nauen and Stumpf [49]. In a black‐walled, clear flat‐bottom 96‐well microplate, 100 μL of monochlorobimane (500 μM), 40 μL of PBS (pH 7.4), and 10 μL of cell‐free extract were added per well. The enzymatic reaction was initiated by adding 100 μL of reduced glutathione (GSH, 0.1 mM). Fluorescence intensity was measured at 460 nm (excitation: 355 nm) at the start of the reaction and then every minute for 5 min. GSH was used as standard. GST activity was calculated as the rate of GSH conversion per minute and expressed as μmol min^−1^ g^−1^ of protein.

#### 2.4.2 Host Biovolume and Parasite Fitness Proxies

Total biovolume was used to standardize biomarker concentrations across treatments. Chytrid biomass represented < 1% of the total culture biovolume (0.7% ± 0.2%, Table S1). Therefore, all measured macromolecules and enzymatic activities were attributed to cyanobacteria. This approach ensured consistent expression of biomarker data across treatments, while acknowledging potential minor contributions from chytrid biomass.

Cyanobacterial biovolume was quantified using an inverted microscope (Nikon Ti Eclipse). Filament volume was estimated by measuring the filament length in 10 fields of a Sedgewick Rafter chamber per individual sample and using the following formula:

$$V=\pi r^{2}h$$

where *r* is the mean filament radius (mean width divided by 2), and *h* is the mean filament length. The mean filament radius was calculated for each exposure treatment (control, MET, and CB leachate) and condition (uninfected and infected) by measuring the width of 10 randomly selected filaments per sample. Dead filaments resulting from chytrid infection (i.e., empty, translucent filaments) were excluded.

Total chytrid biovolume was calculated as the sum of sporangia and zoospore volumes, quantified using a Sedgewick Rafter chamber under an inverted microscope (Nikon Ti Eclipse). Sporangia volume was measured in the same fields used for cyanobacterial biovolume estimation. Empty sporangia were excluded from chytrid biovolume calculations. Sporangia volume was estimated as rotational ellipsoids:

$$V=\pi /6\times d_{1}^{2}\times d_{2}$$

where *d*_*1*_ and *d*_*2*_ are the short and long semi‐axes, respectively. When more than six sporangia were present on the same side of a host cell, they were quantified as a single ellipsoid, as determining the exact volume of overlapping sporangia poses a challenge.

Zoospores volume was determined by quantifying the zoospore density in the same fields used for cyanobacterial biovolume measurements. Based on unpublished data, each zoospore was estimated to have an average volume of 15.5 μm^3^.

Parasite fitness proxies additionally included: infection prevalence and infection intensity. Infection prevalence indicates the proportion of infected filaments within a host population and was calculated as the percentage of cyanobacterial filaments containing encysted zoospores or sporangia after randomly examining 200 filaments under an inverted microscope (Nikon Ti Eclipse). Infection intensity represents the number of parasites infecting a single host. It was determined as the average number of parasites (encysted zoospores or sporangia) per infected filament after randomly examining 50 infected filaments. When more than six infections were present on the same side of a single host cell, infected filaments were categorized as having six infections due to counting limitations.

#### 2.4.3 Chemical Analyses of MET, Nicotine, and Cotinine

MET concentrations were measured to confirm initial levels and stability throughout the experiment. Because CB leachate is a complex mixture of numerous chemicals, assessing the concentration and stability of individual compounds is challenging. Therefore, we chose nicotine, a major organic compound in CBs, and its metabolite, cotinine, as indicators of leachate stability. While these two compounds provide valuable information on leachate dynamics, we recognize that they may not fully represent the stability of all components present in CB leachate.

MET, nicotine, and cotinine were quantified using liquid chromatography electrospray ionization tandem mass spectrometry (LC‐ESI‐MS/MS) with an Agilent 1290 Infinity II UHPLC system coupled to an Agilent 6470 triple quadrupole mass spectrometer in positive ionization mode, following the previously described methods [33, 34].

### 2.5 Data Analyses

To integrate biomarker results and evaluate the global response of the host under multiple stressors, we calculated the IBRv2 following the method described by Sanchez et al. [40]. IBRv2 combines multiple biomarker responses into a single index, allowing assessment of the overall physiological stress experienced by an organism. By incorporating oxidative stress and macromolecular responses, IBRv2 provides a measure of global metabolic disturbance that complements analyses of individual biomarkers. The IBRv2 calculation compares individual biomarker values to a reference mean, corresponding, in our study, to the mean biomarker values of non‐exposed cultures (for both uninfected and infected conditions, respectively). To preserve individual data variability, we included biomarker values from all five replicates of the control, MET‐exposed, and CB leachate‐exposed treatments. For the IBRv2 analysis of uninfected cultures, the extracellular MC‐X values were excluded due to the majority of samples being below the detection limit, which resulted in numerous zero values that were incompatible with the IBRv2 analysis. For the infected cultures, the extracellular DMC‐LR and MC X were excluded for the same reason. IBRv2 values closer to those of the unexposed group indicate a more similar physiological state, while values that deviate further from the control group represent a more divergent response relative to the control condition. Biomarker values are displayed in a star plot, where positive values indicate biomarker increase and negative values indicate biomarker inhibition relative to the control.

Linear models were used to assess the effects on parasites' fitness, biomarker responses, and IBRv2. Cyanobacterial biovolume was analyzed with a linear mixed model to account for repeated measurements within the same sample. A two‐way interaction was included in the IBRv2 model to test for interactions between treatment (control, MET, or CB leachate) and condition (uninfected and infected). The significance of this interaction was assessed by comparing a model with the interaction term to one without it.

Residuals distributions were visually examined to ensure normality and homogeneity of variance. If assumptions were not met, data were log‐ or square root‐transformed. Homoscedasticity of residuals was tested with the Breusch Pagan test. When transformations failed to meet assumptions, non‐parametric linear models were applied.

### 2.6 Software for Data Processing, Analysis, and Visualization

Biovolume measurements were performed using the NIS‐Elements BR 4.5 software (Nikon). Statistical analyses and figures were carried out in R Statistical language (version 4.2.3; [50]), using the packages described below. Linear models were run using base R, linear mixed models were run using *lme4* [51], and post hoc tests were conducted with *emmeans* [52]. Data visualization for normality and homogeneity of variance was done with *ggResidpanel* [53], while the Breusch‐Pagan test was performed using *lmtest* [54]. Conditional and marginal *R* ^2^ for mixed models were estimated according to Nakagawa and Schielzeth [55] with *MuMIn* [56]. IBRv2 was calculated using the *IBRtools* package [57]. Figures were generated with *ggplot* [58], *ggpubr* [59], *cowplot* [60], and *fmsb* [61]. Additionally, various *tidyverse* packages [62] were used for data import, export, tidying, and organization. All R code is provided in the Supporting Information.

## 3 Results

### 3.1 Biomarkers

Protein and lipid concentrations in cyanobacteria exposed to CB leachate alone were significantly lower than in the control (proteins: adj. *R* ^2^ = 0.40, *p* = 0.0161; lipids: adj. *R* ^2^ = 0.47, *p* = 0.0068; Figure 1A,B; Table S2), while MET exposure had no effect. In cultures exposed to both chemical stressors (MET or CB leachate) and parasites, protein and lipid levels were comparable across treatments.

**FIGURE 1:**
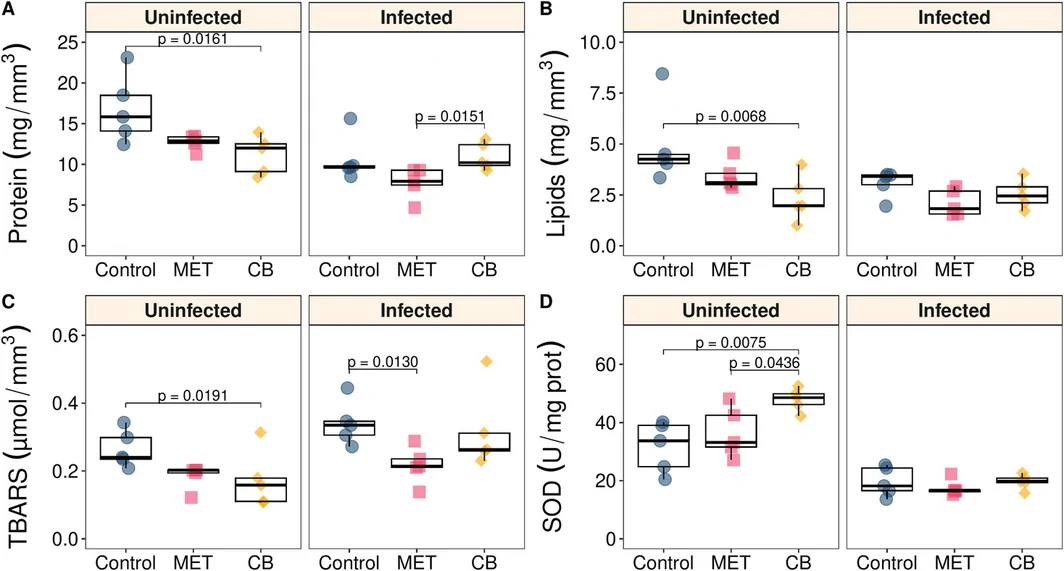
Biochemical parameters and oxidative stress markers in cyanobacteria exposed to chemical pollutants with and without chytrid parasites. (A) Protein concentration, (B) lipid concentration, (C) thiobarbituric reactive substances (TBARS) concentration, and (D) superoxide dismutase (SOD) activity. Protein, lipid and TBARS concentrations were adjusted to the total biovolume of each replicate per treatment and condition. SOD activity was adjusted to the protein content of each replicate per treatment and condition. Boxes indicate upper and lower quartiles, the dark middle line indicates the median, whiskers indicate 1.5 times the interquartile range and black points represent outliers (*n* = 5). CB, cigarette butt leachate; MET, metolachlor.

TBARS levels in uninfected cultures were unaffected by MET but significantly decreased under CB leachate compared to the control (adj. *R* ^2^ = 0.41, *p* = 0.0191; Figure 1C; Table S2). In infected cultures, MET exposure significantly reduced TBARS levels relative to the control (adj. *R* ^2^ = 0.42, *p* = 0.0130), while CB leachate had no effect (Figure 1C; Table S2).

Three MC variants were detected in *P. agardhii* strain NIVA‐CYA630: DMC‐RR (90%–95% of total MCs), DMC‐LR (4%–9%), and an unidentified variant, MC‐X (< 1%) (Table S3). Total and intracellular DMC‐RR and DMC‐LR concentrations followed similar patterns (Figure 2 and Figure S4). In uninfected cultures, MET exposure did not affect their concentrations, whereas CB leachate significantly reduced them (adj. *R* ^2^ = 0.34–0.42, *p* = 0.0151–0.0304, Table S4). Conversely, in infected cultures, MET exposure significantly decreased total and intracellular DMC‐RR and DMC‐LR concentrations relative to the control (adj. *R* ^2^ = 0.48–0.55, *p* = 0.0045–0.0258), while CB leachate had no effect (Table S4).

**FIGURE 2:**
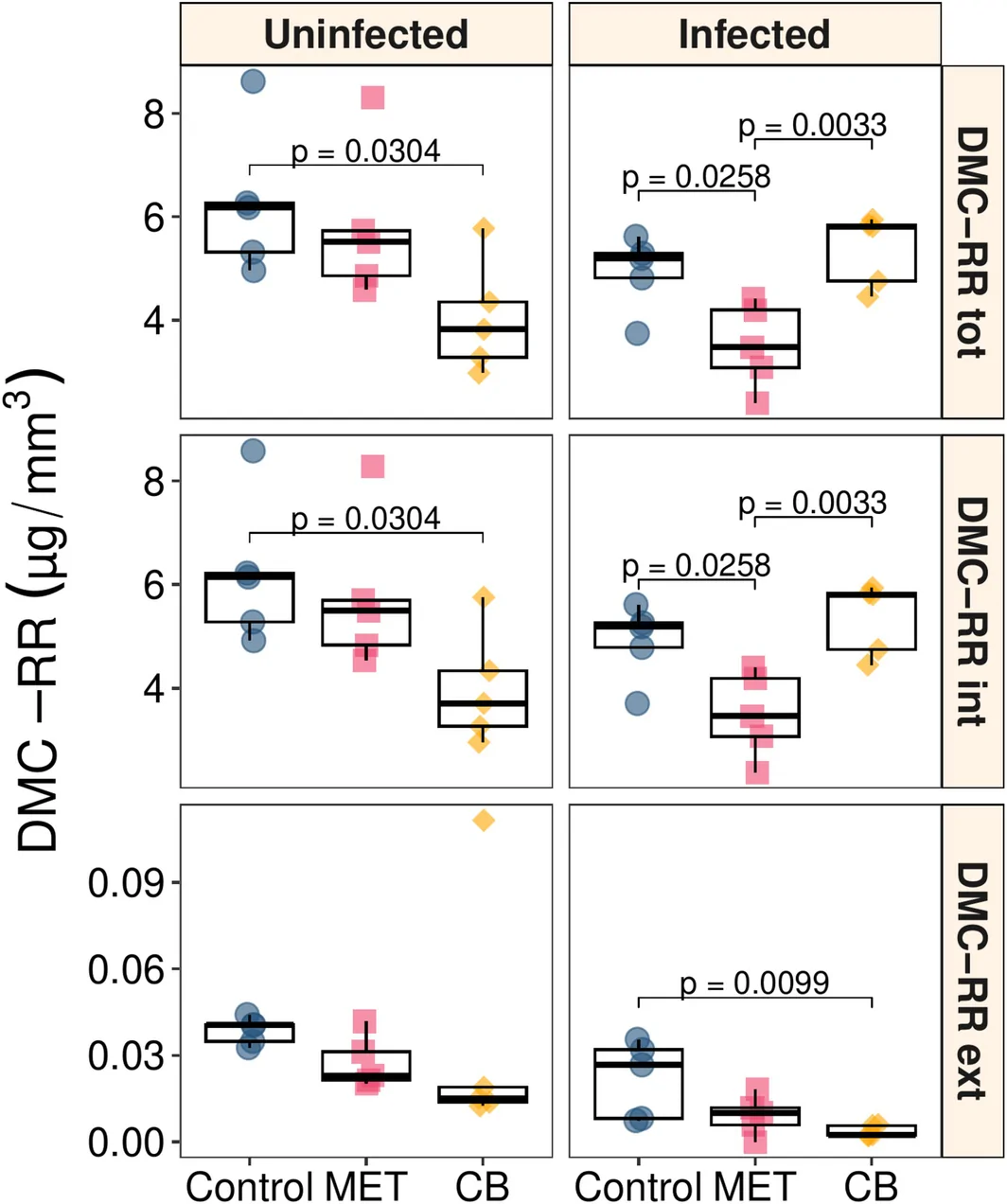
Total, intracellular and extracellular DMC‐RR concentrations in cyanobacteria exposed to chemical pollutants with and without chytrid parasites. DMC‐RR concentrations were adjusted to the cyanobacterial biovolume of each replicate per treatment and condition. Boxes indicate the upper and lower quartiles, the dark middle line indicates the median, whiskers indicate 1.5 times the interquartile range and black points represent outliers (*n* = 5). CB, cigarette butt leachate; MET, metolachlor.

Extracellular DMC‐RR and DMC‐LR levels in uninfected cultures were consistent across treatments (Figure 2 and Figure S1). In infected cultures, CB leachate significantly reduced extracellular DMC‐RR (DMC‐RRe Control‐CB: adj. *R* ^2^ = 0.44, *p* = 0.0099; Table S4), and both CB leachate and MET exposure reduced extracellular DMC‐LR relative to the control (DMC‐LRe Control‐CB and Control‐MET: adj. *R* ^2^ = 0.41, *p* = 0.0300; Figure 2 and Figure S1; Table S4).

Total and intracellular MC‐X concentrations were significantly lower in uninfected cultures exposed to CB leachate compared to the control (MC‐Xt‐i Control‐CB: adj. *R* ^2^ = 0.47, *p* = 0.0098; Figure S2; Table S4), but no differences were observed in infected cultures. Extracellular MC‐X concentrations were consistent across treatments regardless of infection (Figure S2; Table S4).

SOD activity in uninfected cultures decreased significantly under CB leachate exposure compared to the control (adj. *R* ^2^ = 0.48, *p* = 0.0075, Figure 1D; Table S2) but was unaffected by MET. In infected cultures, SOD activity was similar across all treatments. GST activity remained consistent regardless of parasite presence or exposure to MET or CB leachate (Figure S3, Table S2).

#### 3.1.1 IBRv2

Star plots of the A index for each biomarker, showing deviation from the control, are presented in Figure 3A,B. In uninfected cultures, most biomarkers in MET‐exposed cyanobacteria were close to control values, whereas those from CB leachate‐exposed cultures showed greater deviations, primarily indicating biomarker reductions (Figure 3A). In contrast, in infected cultures, MET exposure resulted in larger deviations, mostly reductions, while biomarkers in CB leachate‐exposed cultures remained close to control (Figure 3B).

**FIGURE 3:**
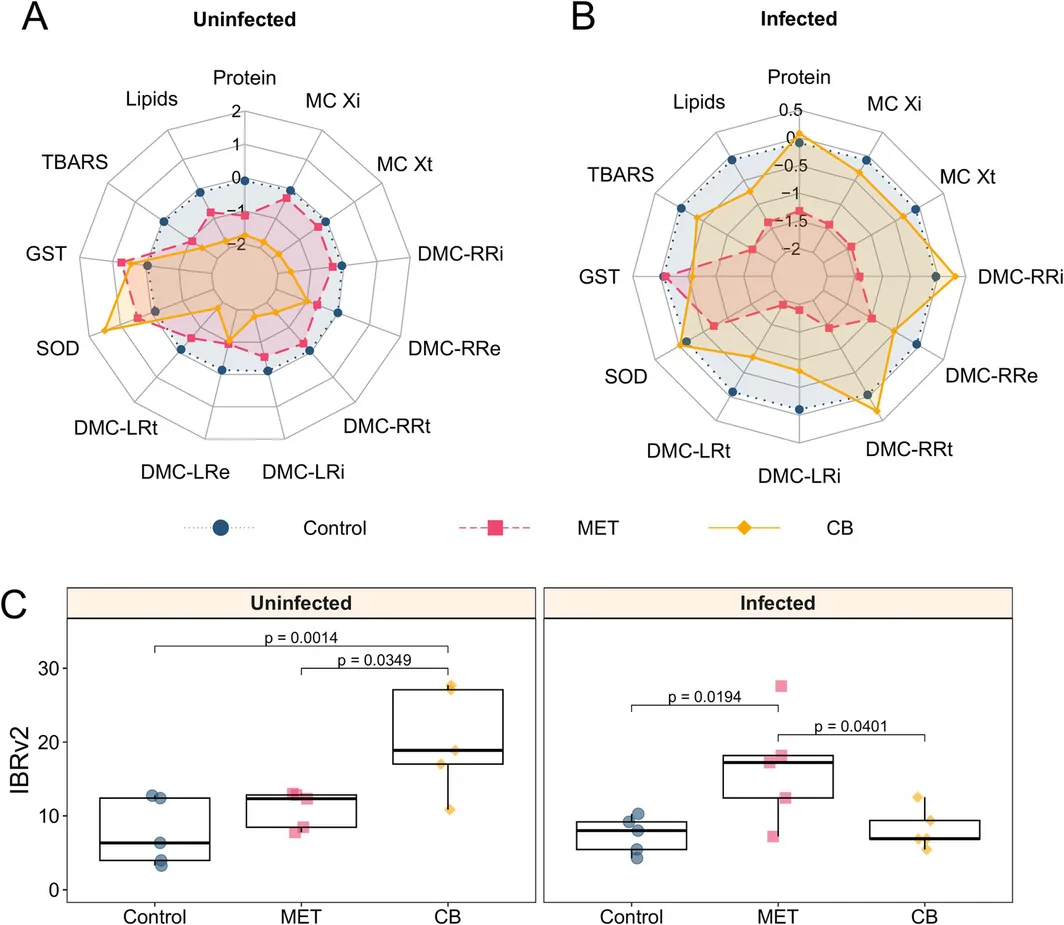
Integrated Biomarker Response version 2 (IBRv2) in cyanobacteria exposed to chemical pollutants with and without chytrid parasites. Star plot of biomarker deviation index of (A) uninfected and (B) infected cultures; (C) comparison of IBRv2 values between treatments. In C, boxes indicate the upper and lower quartiles, the dark middle line indicates the median, whiskers indicate 1.5 times the interquartile range and black points represent outliers (*n* = 5). CB, cigarette butt leachate; GST, glutathione *S*‐transferase; MC I, intracellular microcystin; MC e, extracellular microcystin; MC t, total microcystin; MET, metolachlor; SOD, superoxide dismutase; TBARS, thiobarbituric acid reactive substances.

IBRv2 values indicate how closely treated cultures resemble control. In uninfected cultures, IBRv2 values were comparable to the control with MET exposure but significantly higher with CB leachate (adj. *R* ^2^ = 0.45, *p* = 0.0014; Figure 3C; Table S5). In infected cultures, IBRv2 values were significantly higher than the control when exposed to MET (adj. *R* ^2^ = 0.45, *p* = 0.0194; Figure 3C; Table S5), but similar to treatments with CB leachate. The difference in IBRv2 between uninfected and infected cultures exposed to CB leachate was significant (adj. *R* ^2^ = 0.45, *p* = 0.0012), suggesting distinct responses depending on parasite presence.

### 3.2 Host Biovolume and Parasite Fitness Proxies

In uninfected cultures, cyanobacterial biovolume increased slightly under MET and CB leachate exposure, but the effect was not significant (Figure S5A). In infected cultures, biovolume was significantly higher under MET exposure compared to the control (*p* = 0.0354), while CB leachate had no effect. Treatment (MET or CB leachate) accounted for 16% of the total variance in the model (conditional *R* ^2^‐marginal *R* ^2^; Table S6).

Infection prevalence was significantly lower in cultures exposed to CB leachate compared to control and MET‐exposed cultures, indicating that CB leachate hindered chytrid infection (adj. *R* ^2^ = 0.65; Control‐CB: *p* = 0.0047; MET‐CB: *p* = 0.0009; Figure S5B, Table S2). However, infection intensity was similar across treatments (Figure S5C, Table S2).

### 3.3 Chemical Analyses of MET, Nicotine, and Cotinine

Measured MET concentrations at the start and end of the experiment confirmed its stability in both the uninfected and infected cultures (~100 μg L^−1^), indicating no substantial abiotic or biotic transformation under the tested conditions (adj. *R* ^2^ = 0.32; uninfected *p* = 0.0834, infected *p* = 0.0310; Figure S4A; Table S5). In the CB leachate treatment (equivalent to 1 CB L^−1^), nicotine concentrations decreased slightly (adj. *R* ^2^ = 0.42; uninfected *p* = 0.0109, infected *p* = 0.0146), while cotinine increased (adj. *R* ^2^ = 0.81; uninfected *p* = 0.0146, infected *p* < 0.0001), indicating partial nicotine transformation to cotinine (Figure S4B,C, Table S5). For simplicity, the Discussion section refers to either the target MET concentration or the calculated CB leachate concentration.

## 4 Discussion

Our results show that simultaneous exposure of the cyanobacterium *P. agardhii* to chemical and biological stressors produces distinct metabolic responses depending on the chemical type. Exposure to an environmentally relevant concentration of MET did not affect biomarkers in uninfected cyanobacteria. However, co‐exposure to MET and chytrids reduced TBARS and MC levels in infected cultures, suggesting intensified metabolic stress. Conversely, CB leachate alone decreased biomolecule concentrations and enzymatic activity in uninfected cyanobacteria but had no effect on infected cultures, suggesting that chytrid parasites mitigate the metabolic stress induced by CB‐released chemicals. These findings highlight how single pollutants (MET) and complex chemical mixtures (CB leachate) can produce distinct metabolic responses when combined with biological stressors such as chytrid parasites. Overall, this study extends our previous work on growth and infection outcomes by revealing underlying metabolic mechanisms.

Protein and lipid content remained unaffected by MET in both culture types. Reports on MET effects in non‐target organisms are inconsistent, with both decreases [63] and increases [64] observed. MET's known inhibition of fatty acid synthesis [65] was not reflected in our results of total lipid levels. Instead, MET may alter specific fatty acids rather than overall lipid content. For instance, MET changes the concentrations of two polyunsaturated fatty acids (PUFAs), palmitic (16:0), and linoleic (18:2(n‐6)) acids in the freshwater diatom *Gomphonema gracile* [66]. These two fatty acids are also present in *P. agardhii* and may play a role in chytrid metabolism, as they have been detected in chytrid zoospores following infection of *P. agardhii* [12]. Thus, lipid composition changes may not affect the growth of uninfected cyanobacteria, supporting our previous observations [33], but in infected cyanobacteria, such changes could indirectly limit nutrient availability for chytrids, explaining the reduced parasite fitness previously observed under prolonged MET exposure [33]. Further studies are needed to determine MET's impact on lipid composition in cyanobacteria.

In contrast, CB leachate reduced protein and lipid levels only in uninfected cyanobacteria, suggesting that chytrids mitigate its impact. Similar reductions in plasma protein content have been observed in Nile tilapia exposed to CB chemicals [39]. These findings align with our earlier results showing that chytrid infection alleviates the negative impact of CB leachate on *P. agardhii* growth [34]. While lipid accumulation is a common stress response in aquatic organisms [46, 67, 68, 69], the observed reduction indicates a disruption of lipid biosynthesis or energy storage. Given that proteins and lipids are essential for metabolism, structural integrity, energy storage and cell‐to‐cell communication, their depletion likely compromises cyanobacterial survival, growth, and adaptability [39, 67, 70]. This mechanistic understanding aligns with our previous observation that CB leachate inhibited cyanobacterial growth in the absence of chytrids [34].

Total and intracellular MC and TBARS concentrations were unaffected by MET in uninfected cyanobacteria, contrasting with studies reporting pollutant‐induced MC increases in *Microcystis aeruginosa* [37, 38, 46, 71]. In contrast, co‐exposure to MET and chytrids decreased MC levels. MCs are known to scavenge reactive oxygen species (ROS) and can decline under high ROS [72, 73]. Additionally, exposure to MET and chytrids reduced TBARS, suggesting decreased lipid peroxidation. Considering that MET may reduce PUFA levels (the primary lipid type affected by lipid peroxidation [74]) and that chytrid infections can destabilize cyanobacterial membranes, the lower TBARS concentrations likely result from both: reduced PUFA availability and decreased membrane surface area available for oxidation. The simultaneous reduction in MCs and TBARS, alongside unchanged SOD and GST activity, suggests that non‐enzymatic antioxidants counteract the oxidative stress induced by MET and chytrids. Together with the macromolecule patterns, these responses may explain why MET‐induced growth promotion disappears in infected cultures [33].

CB leachate decreased total and intracellular MCs and TBARS while increasing SOD activity only in uninfected cultures. These results suggest that CB leachate induces oxidative stress in the absence of the parasite, countered by both enzymatic and non‐enzymatic antioxidant mechanisms. However, in co‐exposed cultures, these biomarkers were unaffected, suggesting parasite‐mediated mitigation of chemical stress. This provides a mechanistic explanation for our earlier observation that chytrids enable *P. agardhii* to maintain growth under CB leachate exposure [34]. Parasites may reduce host stress either by bioaccumulating pollutants [75, 76] or by enhancing host resistance to pollutants through metabolic alterations [77, 78]. Thus, co‐exposure to CB leachate and chytrids lowers stress levels in cyanobacteria compared to exposure to the chemical stressor alone.

Cyanobacterial biovolume remained unchanged in uninfected MET‐exposed cultures but increased in infected ones. This contrasts with previous findings where continuous MET (100 μg L^−1^) exposure promoted cyanobacterial growth, but this effect was reversed by chytrids [33]. This discrepancy likely reflects the shorter experimental duration (5 vs. 7 days) and the single‐time‐point in this study, whereas multiple time points in the previous work, captured growth dynamics and cumulative effects more effectively. At 5 days, infection levels were already high, but mortality had not yet occurred. Meanwhile, CB leachate (1 CB L^−1^) had no effect on cyanobacterial biovolume in either culture type, likely due to the lower concentration used here compared with other studies [34, 79, 80]. None of the chemicals altered infection intensity (i.e., the number of parasites per host), but CB leachate reduced infection prevalence. This supports previous findings that chemical stress can impair the chytrid infection cycle, resulting in lower infection prevalence without affecting infection intensity [34, 81].

IBRv2 results further confirm contrasting interactions. Co‐exposure to MET and chytrids increased IBRv2 values, indicating a synergistic effect. Together with our previous finding that long‐term MET exposure decreases chytrid fitness, these results suggest that MET may indirectly impact parasites by altering host nutrient composition [33]. In contrast, CB leachate increased IBRv2 values only in uninfected cultures, suggesting an antagonistic interaction in which parasites enhance host tolerance to chemical stress. This aligns with earlier observations showing cyanobacterial growth inhibition by CB leachate in the absence of parasites, but enhanced tolerance when chytrids were present [34]. Given the complex composition of CB leachate, the observed effects cannot be attributed to individual components and are therefore interpreted as the combined outcome of the chemical mixture.

Overall, our results support all four hypotheses. First, MET did not alter the biomarker responses of uninfected cyanobacteria, indicating low stress in the presence of this chemical. Second, co‐exposure to MET and chytrids synergistically increased cyanobacterial stress, evidenced by the elevated integrated biomarker response. Third, CB leachate induced stress in cyanobacteria, evidenced by altered oxidative stress and macromolecule markers. Finally, chytrid infection mitigated CB‐leachate induced stress, evidenced by the decrease in the integrated biomarker response under co‐exposure.

## 5 Conclusions

Our study highlights the complexity of cyanobacterial responses to simultaneous abiotic (chemical) and biotic (chytrid parasites) stressors, providing mechanistic insights into cyanobacterium‐chytrid interactions under chemical exposure. Chytrid parasites can modulate host stress responses, ultimately influencing the impact of pollutants on cyanobacteria and potentially influencing broader aquatic ecosystem processes. These results emphasize the importance of incorporating biotic interactions, particularly host–parasite dynamics, into ecotoxicological assessments to better understand the effects of pollution on ecosystem health. Moreover, complementing growth‐based toxicity assessments with biomarker analyses offers a more comprehensive understanding of stress mechanisms and organismal responses to pollution.

## Author Contributions

**Erika Berenice Martínez‐Ruiz:** conceptualization, methodology, investigation, formal analysis, resources, writing – original draft, writing – review and editing, visualization, supervision, project administration, funding acquisition. **Jutta Fastner:** methodology (microcystin quantification), resources, writing – review and editing. **Stephanie Spahr:** methodology (chemical analyses), resources, writing – review and editing. **Justyna Wolinska:** resources, writing – review and editing.

## Funding

This work was supported by Deutsche Forschungsgemeinschaft (MA 9934/1‐1) and HORIZON EUROPE Marie Sklodowska‐Curie Actions (101120280).

## Conflicts of Interest

The authors declare no conflicts of interest.

## Supporting information


**Data S1:** Supporting information.

**Data S2:** Supporting information.

## Data Availability

The data and code supporting this study are available as Supporting Information at Zenodo (https://doi.org/10.5281/zenodo.18774139).
